# SARS-CoV-2 detection in the lower respiratory tract of invasively ventilated ARDS patients

**DOI:** 10.1186/s13054-020-03323-5

**Published:** 2020-10-16

**Authors:** Niccolò Buetti, Paul-Henri Wicky, Quentin Le Hingrat, Stéphane Ruckly, Timothy Mazzuchelli, Ambre Loiodice, Pierpaolo Trimboli, Valentina Forni Ogna, Etienne de Montmollin, Enos Bernasconi, Benoit Visseaux, Jean-François Timsit

**Affiliations:** 1 University of Paris, INSERM, IAME, F-75006 Paris, France; 2 Ente Ospedaliero Cantonale, Locarno Community Hospital, Locarno, Switzerland; 3 Infection Control Program and WHO Collaborating Centre on Patient Safety, University of Geneva Hospitals and Faculty of Medicine, Geneva, Switzerland; 4 Medical and Infectious Diseases ICU (MI2), Bichat-Claude Bernard Hospital, AP-HP, 75018 Paris, France; 5 AP-HP, Hôpital Bichat-Claude Bernard, Laboratoire de virologie, F-75018 Paris, France; 6 Faculty of Biomedical Sciences, Università della Svizzera Italiana (USI), Lugano, Switzerland; 7 Clinic of Endocrinology and Diabetology, Lugano and Mendrisio Regional Hospital, Ente Ospedaliero Cantonale, Lugano, Switzerland; 8 Ente Ospedialiero Cantonale, Division of Infectious Diseases, Regional Hospital Lugano, Lugano, Switzerland

**Keywords:** SARS-CoV-2, COVID-19, Viral shedding, Viral load, Lower respiratory tract, ICU, Mortality

## Abstract

**Background:**

Data on SARS-CoV-2 load in lower respiratory tract (LRT) are scarce. Our objectives were to describe the viral shedding and the viral load in LRT and to determine their association with mortality in critically ill COVID-19 patients.

**Methods:**

We conducted a binational study merging prospectively collected data from two COVID-19 reference centers in France and Switzerland. First, we described the viral shedding duration (i.e., time to negativity) in LRT samples. Second, we analyzed viral load in LRT samples. Third, we assessed the association between viral presence in LRT and mortality using mixed-effect logistic models for clustered data adjusting for the time between symptoms’ onset and date of sampling.

**Results:**

From March to May 2020, 267 LRT samples were performed in 90 patients from both centers. The median time to negativity was 29 (IQR 23; 34) days. Prolonged viral shedding was not associated with age, gender, cardiac comorbidities, diabetes, immunosuppression, corticosteroids use, or antiviral therapy. The LRT viral load tended to be higher in non-survivors. This difference was statistically significant after adjusting for the time interval between onset of symptoms and date of sampling (OR 3.78, 95% CI 1.13–12.64, *p* = 0.03).

**Conclusions:**

The viral shedding in LRT lasted almost 30 days in median in critically ill patients, and the viral load in the LRT was associated with the 6-week mortality.

## Background

The SARS-CoV-2 disseminated in Europe in late February 2020, causing the largest pandemic due to any respiratory viruses any respiratory viruses in recent history [1]. Several authors suggested that viral shedding and severity of disease might be correlated [2], but they mostly focused on viral presence in upper respiratory secretions [3, 4]. Viral shedding from upper respiratory tract appeared to be higher soon after symptoms’ onset, but during the course of disease, the shedding originates predominantly from the lower respiratory tract (LRT) [5]. To date, data on viral replication in distal airways are scarce. Only one small study partly investigated the role of viral presence into LRT [6]. Moreover, the association between SARS-CoV-2 viral load in LRT and mortality remains unevaluated [7]. Our objectives were (1) to describe the viral shedding and the viral load in LRT and (2) to determine THE ASSOCIATION BETWEEN VIRAL PRESENCE AND MORTALITY in critically ill COVID-19 patients.

## Material and methods

### Study design and population

We conducted a binational study merging prospectively collected data from two COVID-19 reference centers in France and Switzerland. We included all COVID-19 patients under mechanical ventilation who underwent regularly to LRT sampling for SARS-CoV-2. The Bichat University Hospital served as a reference center for the Northern Paris Region, whereas the Locarno Regional Hospital was an entirely COVID-19-dedicated hospital for all (*n* = 7) public hospitals of southern Switzerland (Canton of Ticino).

### Data collection

All data of COVID-19 patients were prospectively collected in two specific standardized institutional databases. We collected demographic characteristics, medical history, characteristics at hospital and intensive care unit (ICU) admissions, organ support procedures, administration of antivirals and other agents, and biological and virological results. Of note, prescription of antivirals and anti-inflammatory drugs varied among countries, availability, and choice of physician.

### Study procedures

All patients had a SARS-CoV-2 positive nasopharyngeal swab before ICU admission. During the ICU stay, all patients underwent a regularly monitoring of LRT samples (LRTS) until the detection of two consecutive negative real-time polymerase chain reaction (RT-PCR) results [8]. All patients underwent regularly to LRT RT-PCR re-evaluation until removal of the endotracheal tube or death. Endotracheal aspirates, bronchoalveolar leakage fluid, and plugged telescoping catheter were all considered as LRTS. The sampling was performed according to a predefined protocol. All specimens were sent to two accredited reference virology laboratories and used for RNA extraction and amplification by RT-PCR techniques utilizing validated commercial kits that were previously described [9]. A cycle threshold (Ct) value of < 40 was defined as positive for SARS-CoV-2 RNA and ≥ 40 was defined as negative (see supplementary material). Then, RT-PCR values were transformed in viral load as previously published [10]. The French and the regional Swiss Ethics Committees approved this study.

### Definitions and objectives

The primary objective was the description of viral shedding duration (i.e., time to negativity) in LRTS and was defined as the time between onset of symptoms and viral clearance process (i.e., first negative detection of viral RNA in LRT). Of note, we previously showed that the association of the first negative RT-PCR with a second negative result was very high (i.e., 97%) in LRTS [11]. Secondary objectives were (1) the analysis of RT-PCR viral load in LRTS and (2) the assessment of 6-week mortality. Patients discharged before 6-weeks were considered alive.

### Statistical analysis

The statistical plan had three steps. First, we explored the duration of viral shedding in LRT using Kaplan-Meier curves for different clinical relevant patient populations (i.e., age, diabetes, cardiac comorbidities, immunosuppression, and corticosteroids during the ICU stay) which are thought to influence viral shedding in upper respiratory tract samples [2, 12–15]. A log rank test was performed; a specific subgroup had *prolonged* viral shedding if the log rank test between the two groups (e.g., diabetic versus non-diabetic) was statistically significant. Second, we evaluated viral load in LRT during the ICU stay (i.e., quantitative RT-PCT) using graphical and descriptive statistics (i.e., Wilcoxon test) between survivors and non-survivors. Third, we determined the association between viral presence in LRT and mortality using mixed-effect logistic models for clustered data (PROC GLIMMIX of SAS) and adjusting for the time between symptoms’ onset and date of sampling. All collected samples were considered for this analysis. These models take into account the clustering effect of multiple sampling per patient and the center effect (i.e., random effect). We performed two sensitivity analyses: the first using only endotracheal aspirates and the second adjusting for sex and age. Tests were two-tailed, with *p* < 0.05 being considered significant. All analyses were performed using SAS (version 9.4) and R (version 3.5.3). The current analysis complied with the STROBE guidelines for observational studies [16].

## Results

From March to May 2020, 267 LRTS were performed in 90 patients from both centers. A median of 3 (interquartile range [IQR] 2; 4) LRTS was collected per patient. Eighty-three percent (*n* = 222) of LRTS were endotracheal aspirates, whereas a bronchoalveolar lavage was performed in 15% (*n* = 41) of samples. The median age was 62.5 years (IQR 54; 70), and 71 (79%) patients were male (Table 1). Thirty-six (40%) patients had a chronic heart failure, 27 (30%) had diabetes mellitus, and 28 (31%) were immunocompromised. The median SAPS II at ICU admission was 45.5 (IQR 37; 61). Eight (9%), 38 (42%), 27 (30%), and 48 (53%) patients received tocilizumab, ritonavir/lopinavir, hydroxychloroquine, and corticosteroids, respectively. Thirty-three patients (26%) died within 6 weeks after symptoms’ onset. The median time to negativity was 29 (IQR 23; 34) days.

**Table 1:** Patients’ characteristics

	Patients (***N*** = 90)
French center (versus Swiss center), *n* (%)	42 (46.7)
Age, median [IQR]	62.5 [54; 70]
Sex, *n* (%)	
Male	71 (78.9)
BMI, median [IQR]	29.6 [26; 33.3]
SAPS II at ICU admission, median [IQR]	45.5 [37; 61]
SOFA at ICU admission, median [IQR]	7 [6; 10]
Comorbidities	
Cardiovascular, *n* (%)	36 (40)
Chronic respiratory failure, *n* (%)	18 (20)
Renal failure, *n* (%)	14 (15.6)
Immunosuppression, *n* (%)	28 (31.1)
Diabetes mellitus, *n* (%)	27 (30)
Cancer, *n* (%)	4 (4.4)
Treatment administered	
Ritonavir/lopinavir, *n* (%)	38 (42.2)
Remdesivir, *n* (%)	8 (8.9)
Hydroxychloroquine, *n* (%)	27 (30)
Tocilizumab, *n* (%)	8 (8.9)
Corticosteroids, *n* (%)	48 (53.3)
Number of different antibiotics utilized (per patient), median [IQR]	7 [4; 10]
Number of bacteremia per patient, mean (SD)	0.80 (3.92)
Number of VAP per patient, mean (SD)	0.92 (1.05)
Number of LRTS per patient, median [IQR]	3 [2; 4]
Viral load (log10 copies per mL), median [IQR]	3.3 [1.8; 5.2]
Time to negativity in LRTS, median [IQR]	29 [23; 34]
Mortality 6 weeks, *n* (%)	23 (25.6)

*IQR* interquartile range, *BMI* body mass index, *SAPS* Simplified Acute Physiology Score, *SOFA* Sequential Organ Failure Assessment score, *ICU* intensive care unit, *LRTS* lower respiratory tract samples, *SD* standard deviation

**Fig. 1:**
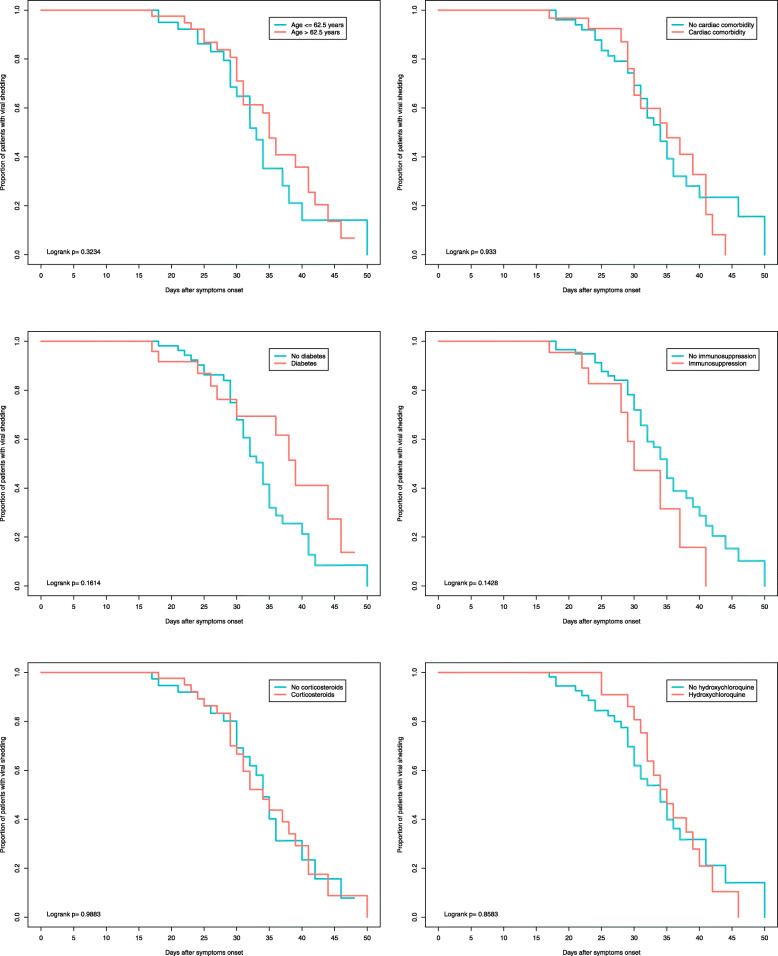
**Viral shedding in lower respiratory tract for the different patient populations, according to patients’ characteristics and to treatments received.** For this descriptive analysis, we performed a log rank test for the different subgroups

Prolonged viral shedding was not associated with age (*p* = 0.32), cardiac comorbidities (*p* = 0.93), diabetes (*p* = 0.16), immunosuppression (*p* = 0.14), nor corticosteroid use (*p* = 0.99, Fig. 1).

**Fig. 2:**
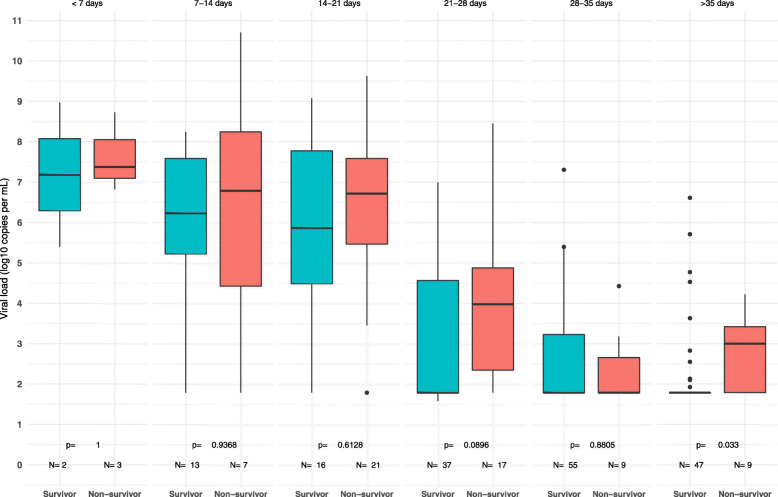
**Viral load among survivors and non-survivors at 6 weeks, stratified by the time between symptoms’ onset and date of sampling.** For this descriptive analysis, we performed a Wilcoxon test between survivors and non-survivors. *N*, number of lower respiratory tract samples performed

However, using a graphical description, the LRT viral load tended to be higher in non-survivors (Fig. 2).

This difference was statistically significant using mixed-effect models and after adjusting for the time interval between onset of symptoms and date of sampling, patient, and center random effect (OR 3.78, 95% CI 1.13–12.64, *p* = 0.03). A sensitivity analysis using only endotracheal aspirates (i.e., without bronchoalveolar leakage fluid and plugged telescoping catheter) showed similar results (OR 2.96, 95% CI 0.78–11.25, *p* = 0.10). After adjustment for age and gender, we observed again similar results (OR 3.25, 95% CI 0.95–11.17, *p* = 0.06).

## Discussion

Our findings have several clinical, pathophysiological, and infection prevention implications. First, we showed that viral load in the LRT was associated with the 6-week mortality. Therefore, positive LRTS may be used as a prognostic marker in COVID-19 patients. Second, COVID-19 is characterized by an initial phase of viral replication in the upper respiratory tract, followed by a second pulmonary phase driven especially by the host inflammatory response [17], and this latter phase probably determines the prognosis of disease. For this reason, some investigators administered immunomodulatory drugs in severe COVID-19 patients [18]. However, our data may suggest a possible direct cytopathic role of virus in the LRT, supported by the significant association between viral load and mortality. Third, we showed that the viral shedding in LRT lasted almost 30 days in median, and we did not identify specific risk factors for prolonged viral shedding in the LRT. Several studies illustrated that viral shedding in the upper respiratory tract in non-critically ill patients varied from 8 to 24 days [2, 5, 12–15, 19]. We observed that the viral shedding in critically ill patients may be longer, probably due to a prolonged replication in the LRT. Interestingly, any of the well-known risk factors for upper respiratory tract appeared to be able to influence the viral shedding in LRT. Critically ill patients may, therefore, require prolonged infection prevention measures during their hospitalization.

Our study has several limitations. First, we performed an observational study using surveillance data and we showed only an association between viral presence and mortality. Second, viral cell cultures (i.e., the gold standard) were not routinely performed during the study: the results on viral shedding might have been different if this method would have been used and firm assumptions on infectivity cannot be made. Third, all aspirates (endotracheal samples, BAL, and telescope catheters) were categorized as LRTS and a comparison between them may be debatable. However, a sensitivity analysis using only endotracheal samples showed similar results. Moreover, the LRTS dilution was not standardized between the two participating centers and a dilution marker was not used. Finally, we did not routinely collect nasopharyngeal samples and the true viral excretion (e.g., from the upper respiratory tract) concomitantly with LRT replication remains unknown; therefore, assumptions on infectivity based on LRT analyses should be interpreted with caution.

## Conclusions

The viral shedding in LRT lasted almost 30 days in median in critically ill patients, and the SARS-CoV-2 viral presence in the LRT was associated with the 6-week mortality.

## Supplementary information

**Additional file 1.**

## Data Availability

The datasets used and/or analyzed during the current study are available from the corresponding author on reasonable request.

## Abbreviations

BMI: Body mass index
Ct: Cycle threshold
ICU: Intensive care unit
IQR: Interquartile range
LRT: Lower respiratory tract
LRTS: Lower respiratory tract samples
RT-PCR: Real-time polymerase chain reaction
SAPS: Simplified Acute Physiology Score
SOFA: Sequential Organ Failure Assessment score

## References

[1] Faust JS, Del Rio C. Assessment of deaths from COVID-19 and from seasonal influenza. JAMA Intern Med. 2020;180(8):1045–46. 10.1001/jamainternmed.2020.2306. 10.1001/jamainternmed.2020.2306 32407441

[2] Zheng S, Fan J, Yu F, Feng B, Lou B, Zou Q (2020). Viral load dynamics and disease severity in patients infected with SARS-CoV-2 in Zhejiang province, China, January-March 2020: retrospective cohort study. Bmj..

[3] Yu F, Yan L, Wang N, Yang S, Wang L, Tang Y, et al. Quantitative detection and viral load analysis of SARS-CoV-2 in infected patients. Clin Infect Dis. 2020;71(15):793–98. 10.1093/cid/ciaa345. 10.1093/cid/ciaa345 PMC7184442 32221523

[4] Fang Z, Zhang Y, Hang C, Ai J, Li S, Zhang W (2020). Comparisons of viral shedding time of SARS-CoV-2 of different samples in ICU and non-ICU patients. J Infection.

[5] Wolfel R, Corman VM, Guggemos W, Seilmaier M, Zange S, Muller MA (2020). Virological assessment of hospitalized patients with COVID-2019. Nature..

[6] Huang Y, Chen S, Yang Z, Guan W, Liu D, Lin Z (2020). SARS-CoV-2 viral load in clinical samples from critically ill patients. Am J Respir Crit Care Med.

[7] Pujadas E, Chaudhry F, McBride R, Richter F, Zhao S, Wajnberg A, et al. SARS-CoV-2 viral load predicts COVID-19 mortality. Lancet Respir Med. 2020;8(9):e70. 10.1016/S2213-2600(20)30354-4. 10.1016/S2213-2600(20)30354-4 PMC7836878 32771081

[8] ECDC (2020) Guidance for discharge and ending isolation in the context of widespread community transmission of COVID-19. Availbale from: https://www.ecdc.europa.eu/sites/default/files/documents/covid-19-guidance-discharge-and-ending-isolation-first%20update.pdf. Last Accessed 15th July 2020. 2020.

[9] Lescure FX, Bouadma L, Nguyen D, Parisey M, Wicky PH, Behillil S (2020). Clinical and virological data of the first cases of COVID-19 in Europe: a case series. Lancet Infect Dis.

[10] Pan Y, Zhang D, Yang P, Poon LLM, Wang Q (2020). Viral load of SARS-CoV-2 in clinical samples. Lancet Infect Dis.

[11] Buetti N, Trimboli PP, Mazzuchelli T, Lo Priore E, Balmelli C, Trkola A, et al. Diabetes mellitus is a risk factor for prolonged SARS-CoV-2 viral shedding in lower respiratory tract samples of critically ill patients. Endocrine. 2020:1–7. 10.1007/s12020-020-02465-4. 10.1007/s12020-020-02465-4 PMC7459254 32870469

[12] Fu Y, Han P, Zhu R, Bai T, Yi J, Zhao X, et al. Risk factors for viral RNA shedding in COVID-19 patients. Eur Respir J. 2020. 10.1183/13993003.01190-2020 PMC7236829 32398298

[13] Xu K, Chen Y, Yuan J, Yi P, Ding C, Wu W, et al. Factors associated with prolonged viral RNA shedding in patients with COVID-19. Clin Infect Dis. 2020;71(15):799–806. 10.1093/cid/ciaa351. 10.1093/cid/ciaa351 PMC7184421 32271376

[14] Xiao AT, Tong YX, Zhang S. Profile of RT-PCR for SARS-CoV-2: a preliminary study from 56 COVID-19 patients. Clin Infectious Diseases. 2020;ciaa460. 10.1093/cid/ciaa460. 10.1093/cid/ciaa460 PMC7188124 32306036

[15] Qi L, Yang Y, Jiang D, Tu C, Wan L, Chen X, et al. Factors associated with duration of viral shedding in adults with COVID-19 outside of Wuhan, China: a retrospective cohort study. Int J Infect Dis. 2020;96:531–37. 10.1016/j.ijid.2020.05.045. 10.1016/j.ijid.2020.05.045 PMC7231495 32425636

[16] von Elm E, Altman DG, Egger M, Pocock SJ, Gotzsche PC, Vandenbroucke JP (2007). Strengthening the Reporting of Observational Studies in Epidemiology (STROBE) statement: guidelines for reporting observational studies. Bmj..

[17] Siddiqi HK, Mehra MR (2020). COVID-19 illness in native and immunosuppressed states: a clinical-therapeutic staging proposal. J Heart Lung Transplantation.

[18] Toniati P, Piva S, Cattalini M, Garrafa E, Regola F, Castelli F (2020). Tocilizumab for the treatment of severe COVID-19 pneumonia with hyperinflammatory syndrome and acute respiratory failure: a single center study of 100 patients in Brescia, Italy. Autoimmunity Reviews.

[19] Yan D, Liu XY, Zhu YN, Huang L, Dan BT, Zhang GJ, et al. Factors associated with prolonged viral shedding and impact of lopinavir/ritonavir treatment in hospitalised non-critically ill patients with SARS-CoV-2 infection. Eur Respir J. 2020. 10.1183/13993003.00799-2020 PMC7241115 32430428

