# Supplementary material for: SARS-CoV-2 detection in the lower respiratory tract of invasively ventilated ARDS patients
Source: Crit Care. 2020 Oct 16;24:610. doi: 10.1186/s13054-020-03323-5 (PMC7562762; doi:10.1186/s13054-020-03323-5)
Supplement: Supplementary file 1 — Additional file 1. [file 13054_2020_3323_MOESM1_ESM.docx]

**Supplementary material: SARS-CoV-2 detection in the lower respiratory tract of invasively ventilated ARDS patients.**

**Methods:**

Virological analyses:

In Switzerland, swab samples were immediately inserted into sterile tubes containing 3 mL UTM-RT® viral transport medium (Copan, Brescia Italy), lower respiratory tract samplings (LRTS) were placed into sterile tubes. LRTS were sent to the microbiology laboratory for sample processing and viral RNA extraction. RNA extracted from LRTS were amplified using a second PCR system based on the protocol published by Corman *et al.* [1] with primers and probes produced by TibMol Biol (Berlin, Germany). The viral load was indicated as cycle threshold (Ct) value of S gene of SARS-CoV-2 (VIASURE) and E- and RdRP gene, respectively. A positive and a negative control, as well as internal controls were included in the assay, according to the manufacturer's protocol. A Ct value of <40 was defined as positive for SARS-CoV-2. RNA and >40 was defined as negative. Samples with a Ct value between 37 to 40, were retested, at least twice.

In France, naso-pharyngeal swab samples were transported into sterile tubes containing 1 mL Virocult viral transport medium (Sigma), while LRTS were placed into sterile tubes and immediately sent to the virology laboratory. Viral RNA were extracted from 200 μL of clinical samples using the MagNA Pure LC Total Nucleic Acid Isolation Kit - Large Volume (Roche Diagnostics) and eluted in 50 μL. A commercial RT-PCR assay (RealStar® SARS-CoV-2) targeting both the E and the S genes of SARS-CoV-2 was performed, according to the manufacturer recommendations. This assay and the WHO assay used in the Swiss reference center have similar limits of detection [2]. A Ct value of <40 was defined as positive for SARS-CoV-2. RNA and >40 was defined as negative.

**Supplemetary references**

1 Corman VM, Landt O, Kaiser M, et al. Detection of 2019 novel coronavirus (2019-ncov) by real-time rt-pcr. *Euro surveillance : bulletin Europeen sur les maladies transmissibles = European communicable disease bulletin*. 2020; **25**.

2 Visseaux B, Le Hingrat Q, Collin G, et al. Evaluation of the realstar(r) sars-cov-2 rt-pcr kit ruo performances and limit of detection. *Journal of clinical virology : the official publication of the Pan American Society for Clinical Virology*. 2020; **129**: 104520.
